# Willingness to be the recipient during the dictator game

**DOI:** 10.1186/s13104-022-06148-3

**Published:** 2022-07-23

**Authors:** Hirofumi Hashimoto, Kaede Maeda, Keisuke Yamamoto, Nobuhiro Mifune

**Affiliations:** 1 Graduate School of Literature and Human Sciences, Osaka Metropolitan University, Osaka, Japan; 2 Department of Psychology, College of Contemporary Psychology, Rikkyo University, Niiza, Japan; 3 Urban-Culture Research Center, Osaka Metropolitan University, Osaka, Japan; 4 School of Economics & Management, Kochi University of Technology, Kochi, Japan

**Keywords:** Dictator game (DG), Role choice, Altruistic allocation, Avoiding decision-making, Strategic consideration

## Abstract

**Objective:**

Researchers have investigated human altruism toward strangers for decades, using economic games such as the dictator game (DG) in their experiments. However, factors that cause the allocating behavior exhibited by those participants willing to be recipients in the DG have not been identified and the psychological mechanism of avoiding decision-making in economic games has not been widely addressed in previous studies. This study aimed to replicate previous findings regarding the number of people who are willing to be assigned the role of recipient and their allocation behavior and to explore why they share more than people who are willing to be dictators.

**Results:**

We demonstrate that there are people willing to be assigned the role of the recipient, rather than the role of the dictator during the dictator game. In addition, we find evidence indicating that people who are willing to be recipients behave more altruistically in the dictator game than those who prefer to be dictators. Based on our results, we argue that willingness to be a recipient, in relation to the psychological unwillingness to assume responsibility and reputational concerns, is a strategic consideration.

**Supplementary Information:**

The online version contains supplementary material available at 10.1186/s13104-022-06148-3.

## Introduction

Why humans display altruism toward strangers who are neither close kin nor unrelated but with whom they repeatedly interact is one of the most profound questions in the social and evolutionary sciences [1]. *Altruism* is generally defined as behaviors that benefit another individual but are costly to the altruist [2]. While various evolutionary theories or models have been proposed [3], many studies have used experimental economic games to answer this question [4]. One such economic game is the dictator game (DG) [5]. In the DG, two individuals are randomly paired and assigned the role of either dictator or recipient. The dictator receives money and is free to decide how much of it is shared with an anonymous recipient. The recipient does nothing during the experiment, and simply accepts the dictator’s decision. If dictators were entirely self-interested, they would give recipients nothing. However, most dictators allocate charitably, even in one-shot, anonymous DGs [6]. A significant factor in allocation behavior in the DG is the social preference for fairness or inequality aversion (e.g., [7, 8]). For example, pro-social individuals with a fairness preference are more likely to allocate than self-interested (i.e., pro-self) individuals (e.g., [9]). Another major factor in allocation behavior is the presence of strategic considerations, for example, concern for reputation [10, 11].

Yamagishi et al*.* [12] reported that the role choice option could be used to distinguish between social preference and strategic consideration in the DG. Their study examined behavioral consistency in various experimental economic games, such as the prisoner’s dilemma and trust game, and the relationship between behavior and preferences. They found that behavior in the DG differed from behavior in the other games because of role choice, as reported by participants after the experiment. Participants preferring the dictator role showed behavioral consistency across games, while participants preferring the recipient role did not. Moreover, participants willing to be recipients gave more money than those willing to be dictators. Thus, the behavior of those willing to be dictators may be regulated by preferences, while that of participants who are willing to be recipients may be regulated by other factors (e.g., strategic consideration). However, Yamagishi et al*.* [12] did not identify which factors caused the allocating behavior exhibited by those participants willing to be recipients.

The current study had two aims. The first objective was to replicate Yamagishi et al.’s results [12]. In their experiment, DG participants made decisions by imagining themselves in the allocator role, and then a subsequent lottery determined whether they assumed the allocator or recipient. Role choice preference was asked exploratorily after the decision was made. Therefore, participants may have indicated willingness to be recipients with the expectation that others would give more. In the present study, we examined how many participants would be willing to be recipients, even in the absence of such an expectation, and tested whether participants who were willing to be recipients gave more money than those willing to be dictators. Our second objective was to explore why participants willing to be recipients give more money than those willing to be dictators.

## Main text

### Methods

Fifty Japanese undergraduate students (21 females and 29 males) participated. Monetary rewards were emphasized as incentives when they were recruited.

When participants arrived at the laboratory, they were individually greeted by a receptionist who gave them 7-digit identity numbers to assure anonymity. Next, each participant was escorted to a private booth. After being seated, participants were given instructions about the DG that explained the aim of the experiment and the rules of the DG. More specifically, it was explained that participants would be randomly paired to play the game and assigned either the dictator or recipient role in their pairs. Then, the dictator, who would be given JPY 900 by the experimenter, could decide on a share of between JPY 0 and JPY 900 for the recipient. Lastly, the recipient should accept the proposer’s decision. After the instructions, participants were asked two questions: one regarding their role choice and one confirming their understanding. After answering the two questions, the experimenter collected the instructions and gave participants a decision-making sheet. In the present study, no actual recipients were involved, and all participants were assigned to the dictator role, making the decision once. After completion of their decision-making in the DG, participants were asked to answer a post-experiment questionnaire. On completing the experiment, participants received the amount of money that they kept from a receptionist who knew nothing about the experiment. Their decision-making was entirely anonymous for the experimenter who interacted with them in person.

Three instruments were used. To identify role choice preference, participants were asked “Which of the two roles would you like to be assigned?” and selected one of four options: “I definitely want to be a dictator,” “I would rather be a dictator,” “I would rather be a recipient,” and “I definitely want to be recipient.” On the decision-making sheet, all participants were informed that they had been assigned the dictator role in a lottery and had to decide how much of the JPY 900 they would keep and how much they would give to their partner (recipient). The 12-item post-experiment questionnaire (see Additional file 1, using a 7-point Likert scale) was designed to investigate what the dictators thought when they decided on the amount of money to offer to the recipient.

## Results

The mean allocation was JPY 257.8 of the JPY 900, and 44.0% (22/50) were equal allocations. Regarding participants’ role choice, results confirmed that 20% preferred being recipients. Moreover, participants who were willing to be recipients allocated more to their recipient (*M* = JPY 416.00) than participants willing to be dictators (*M* = JPY 218.25; *Mann-Whitney U* = 320.00, Z = -3.03, *p* < 0.01, effect size *r* = 0.433, 95% CI [0.179, 0.626]).).

**Fig. 1:**
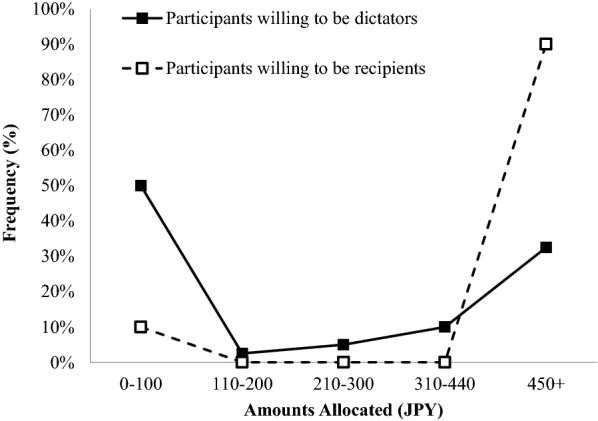
Distributions of the Amounts (JPY) Allocated to the Recipients by Participants’ Role Chice

Figure 1 shows the distributions of the amounts allocated to recipients by participants’ role choice. The distribution of amounts allocated by participants who were willing to be dictators was bimodal, suggesting that both self-interested and other-regarding tendencies existed within this group. However, the distribution of participants who were willing to be recipients showed a pronounced altruistic allocation. Alternatively, this result could indicate that a notable percentage of participants who showed altruistic allocations were willing to be recipients. In our data, 9 of the 22 equal allocators (40.9%) in the DG were willing to be recipients. As a side note, no participants indicated that they definitely wanted to be recipients. In the above analysis, those who indicated that they definitely or would rather be a dictator were integrated, although Additional file 1 demonstrates the distribution by participants’ responses, as a reference.

Because of the small sample size, we examined all single item means and standard deviations (see Additional file 2). Although this was an exploratory analysis, we performed a post hoc analysis of mean differences by role choice to interpret the psychological mechanism behind the willingness to be a recipient. People willing to be dictators gave markedly different ratings than those willing to be recipients on statements such as, “I would feel somewhat bad if my share is more than the recipient’s.” Participants who were willing to be recipients scored higher (*M* = 6.20) than participants willing to be dictators (*M* = 4.15; *t* (48) = 2.73, *p* < 0.01, *d* = 0.95, 95% CI [0.24, 1.66]). This finding could mean that a sense of assuming responsibility increases the amount of money allocated in the DG and leads to a preference for the recipient rather than the dictator role to avoid decision-making or responsibility.

## Discussion

Our results demonstrated that a percentage of people (20%) are willing to be recipients in the DG, and they allocate more to others than those willing to be dictators. Although the percentage of people willing to be recipients was below that in Yamagishi et al.’s [12] findings (36%), the present study’s results were comparable.

Previous studies have not comprehensively addressed the psychological mechanism of avoiding decision-making in economic games. More specifically, interpreting the reason for choosing the recipient role is not straightforward because individuals with a fairness preference would probably be willing to be assigned the dictator rather than the recipient role, as would other-regarding participants, who care not only about themselves but also others’ interests. Hence, it would be difficult to infer the existence of participants with a preference for the recipient role from the self-interested or other-regarding preferences discussed in previous studies.

Exploratory analyses suggested that participants who were willing to be recipients care about the recipient’s feelings and think about their decision. These results indicate two possible strategic considerations that lead to the altruistic allocation exhibited by people willing to be recipients. One consideration is a reputational concern. Dana et al. [10] showed the effect of reputational concern in the DG using an exit option. In their experiment, the dictator was free to decide on a share of the money (USD10) but was additionally given an opportunity to either participate in the DG or withdraw from participating. If the dictator chose to exit, the dictator received USD 9 and the recipient received USD 0, although the recipient was not informed that the DG experiment was conducted, that is, the recipient did not know anything about the DG experiment. Choosing this exit option is similar to the role choice in the current study in that it avoids the giving behavior itself.

The other strategic consideration is about assuming responsibility. Cryder and Loewenstein [13] demonstrated that people behave more altruistically in a DG when they feel responsible for the recipient’s outcome than when they do not. These findings are closely related to a classic social psychology study indicating bystander effects [14], and it seems reasonable to argue that assuming responsibility may manifest itself in altruistic allocation in the DG. People who are willing to be recipients in the DG would likely be willing to avoid the responsibility of determining the recipient’s reward through their own decision. Future studies should examine the validity of these possibilities.

## Limitations

While the present study provides important insights that some people are altruistic and might be willing to avoid the responsibility, several limitations must be addressed. First, the sample size was small. Because of the consistency with Yamagishi et al.’s [12] findings, the generalizability of the findings seems promising. However, replication studies should be conducted to confirm the reproducibility of this phenomenon. A related issue is that willingness to be a recipient might only be observed in Japanese samples. Some research findings demonstrate that the Japanese tend to avoid a negative reputation in social contexts [15, 16] and adopt “not-offend-others-strategies” that meet others’ expectations by default [17–19]. Therefore, only the Japanese might prefer to be recipients in such high proportions, and such preferences may not be observed in other cultures. The level of this response should be compared in cross-cultural research. Second, it is difficult to draw any firm conclusions about the psychological mechanism of being willing to accept the recipient role solely from the present study’s results. Our findings make sense as a description, but not as an explanation. Furthermore, the results of the post-questionnaire are only informative, and do not provide a detailed explanation of why participants preferred to be recipients. Therefore, further research is needed to identify the underlying psychological mechanisms (e.g., trust, cooperation, or indifference) for the preference by carefully examining the response patterns of those willing to be recipients. Third, we should clarify that the role choice we asked for in this study was only imaginary and not incentivized (i.e., the role was not in reality determined according to the participants’ choices), as this study attempted to identify the relationship between altruistic behavior in DG and role preference. However, in future studies, it would be worth considering whether the preference to be recipients, as shown in this study, is also shown in incentivized conditions.

## Supplementary Information

**Additional file 1.** Distributions of the Amounts (JPY) Allocated to the Recipients by Participants’ Role Choice (three options)

**Additional file 2.** Mean score and standard deviation of each item of the post-experiment questionnaire.

## Data Availability

Materials and data for the present study are available on OSF. https://osf.io/6u95m/?view_only=70ac6489668c4c5598de3a0bd195414d.
