# Supplementary material for: Willingness to be the recipient during the dictator game
Source: BMC Res Notes. 2022 Jul 23;15:261. doi: 10.1186/s13104-022-06148-3 (PMC9308284; doi:10.1186/s13104-022-06148-3)
Supplement: Supplementary file 1 — Additional file 1. Distributions of the Amounts (JPY) Allocated to the Recipients by Participants’ Role Choice (three options) [file 13104_2022_6148_MOESM1_ESM.docx]

**Additional file 1.**

*Distributions of the Amounts (JPY) Allocated to the Recipients by Participants’ Role Choice (three options)*
