# Supplementary material for: Willingness to be the recipient during the dictator game
Source: BMC Res Notes. 2022 Jul 23;15:261. doi: 10.1186/s13104-022-06148-3 (PMC9308284; doi:10.1186/s13104-022-06148-3)
Supplement: Supplementary file 2 — Additional file 2. Mean score and standard deviation of each item of the post-experiment questionnaire. [file 13104_2022_6148_MOESM2_ESM.docx]

**Additional file 2.**

| **Items** | **Mean (SD)** | | ***t* value** |
| --- | --- | --- | --- |
|  | **Participants willing to be dictators**  **(*n* = 40)** | **Participants willing to be recipients**  **(*n* = 10)** |  |
| Q. To what extent did you think about the following, when you decided on the amount you shared? | | | |
| 1. **I would feel somewhat bad if my share is more than the recipient’s.** | 4.150 (2.282) | 6.200 (1.229) | **2.729^**^** |
| 1. **I want to share more money with the recipient than I do with myself.** | 1.850 (1.027) | 3.200 (1.687) | **3.239^**^** |
| 1. **I do not care what the recipient thinks about my share; I just want to share as I want.** | 4.400 (2.098) | 2.600 (1.955) | **2.458^*^** |
| 1. **I only want to pursue my own interests.** | 4.075 (2.212) | 2.000 (1.563) | **2.788^**^** |
| 1. Someone is testing me to see how much I am willing to share with the recipient. | 4.750 (2.048) | 5.700 (1.252) | 1.397 |
| 1. **Whether or not the recipient will be happy when he/she finds out how much I shared.** | 3.250 (1.958) | 4.700 (1.160) | **2.235^*^** |
| 1. Whether or not the recipient will be saddened when he/she finds out how much I shared. | 3.550 (1.947) | 4.200 (1.687) | 0.967 |
| 1. **Whether or not the recipient will be angry when he/she finds out how much I shared.** | 3.075 (1.730) | 4.500 (1.650) | **2.349^*^** |
| 1. What allocation the experimenter wants me to make? | 3.350 (2.082) | 4.300 (2.111) | 1.287 |
| 1. I have to act in accordance with the expectations of the recipient. | 2.725 (1.935) | 3.900 (1.287) | 1.815 |
| 1. I want the allocation amount to be different from the experimenter’s expectations. | 2.375 (1.877) | 1.800 (1.549) | 0.894 |
| 1. Since I am participating in an experiment, I will do something that I would not have done otherwise. | 2.500 (2.148) | 1.700 (1.337) | 1.119 |

*Note. N* = 50. Significant differences are indicated in bold font. Participants indicated the extent of their thoughts on a scale from 1 (never thought about it at all) to 7 (very well thought about it).

*^*^ p* < .05. *^**^ p* < .01.
